# *Sepia pharaonis* Ink Mitigates Dehydroepiandrosterone-Induced Insulin Resistance in Mouse Model of Polycystic Ovarian Syndrome

**DOI:** 10.3390/pathophysiology31030031

**Published:** 2024-08-15

**Authors:** Prathyusha Yamarthi, Rama Satyasri Kotipalli, Samatasai Patnaik, Kv Veena, Muralidharan Kathirvel, Rajkumar Vutukuri, Manjula Bhanoori

**Affiliations:** 1 Department of Biochemistry, Osmania University, Hyderabad 500007, India; prathyusha.yamarthi@gmail.com (P.Y.); veenathareesh090@gmail.com (K.V.); 2 Applied Biology, CSIR—Indian Institute of Chemical Technology, Hyderabad 500007, India; satyasri.15247@csiriict.in (R.S.K.); samata.spatnaik@gmail.com (S.P.); muralidharan@iict.res.in (M.K.); 3 Institute of General Pharmacology and Toxicology, Pharmazentrum Frankfurt, Goethe University Frankfurt, 60596 Frankfurt am Main, Germany

**Keywords:** *Sepia pharaonis* ink, PCOS, insulin resistance, DHEA, RT-PCR, metformin

## Abstract

The present study aims to evaluate the effect of *Sepia pharaonis* ink on insulin resistance in PCOS-induced mice. Treatment with sepia ink in dehydroepiandrosterone (DHEA)-induced PCOS mice at various doses of 50 mg/kg, 100 mg/kg, and 200 mg/kg body weight mitigated the insulin resistance in the study groups with decreased concentration of testosterone and increased concentrations of estrogen and progesterone compared to the PCOS group tested by ELISA. The histopathological analysis and restoration of glucose analysis showed a significant reduction in treatment groups. Reduced expression of insulin resistance genes like androgen receptor (AR), insulin receptor substrate 1 (IRS-1), and insulin-like growth factor1 (IGF-1) by qRT-PCR indicate a positive impact of sepia ink in alleviating the symptoms associated with PCOS. Taken together, the results of this study indicate sepia ink as a promising therapeutic intervention and a possible drug target for insulin resistance in diabetes and gynecological disorders like PCOS.

## 1. Introduction

Polycystic ovarian syndrome (PCOS) is a common endocrine disorder affecting women of childbearing age, with a global incidence ranging from 6 to 21% [1]. It is a leading cause of infertility, responsible for 70–80% of infertility cases in women worldwide [2,3]. Recent research suggests that 72% of women with PCOS experience infertility, a significantly higher rate compared to those without the condition [4]. PCOS is characterized by various symptoms as per Rotterdam criteria, including hyperandrogenism, irregular menstrual cycles, ovarian cysts, and metabolic disturbances such as insulin resistance. These symptoms often manifest as acne, hirsutism, weight gain, and mood disorders like anxiety and depression [3,5]. Despite extensive research efforts, its precise etiopathology remains poorly understood, and diagnostic criteria are still considered inadequate. Currently, there exists no definitive cure for PCOS, and treatment primarily involves lifestyle modifications. Additionally, the presence of other metabolic conditions such as obesity, type 2 diabetes, and cardiovascular events complicates the management of the condition [3,4,6]. Given its complexity, addressing PCOS requires a multifaceted treatment approach that targets normalization of ovulation, reduction of androgen levels, and improvement of insulin sensitivity [7].

Metformin, originally used to treat type 2 diabetes, has emerged as a potential treatment for PCOS due to its ability to improve insulin sensitivity and restore hormonal balance, leading to more regular menstrual cycles [8]. However, its efficacy in PCOS treatment remains debated, and its mechanism of action is not fully elucidated [9,10]. In response to the limitations of conventional treatments, there is growing interest in incorporating natural therapies into PCOS management [11]. Many methods are now being used to treat PCOS and stimulate ovulation. However, significant adverse effects have been documented, including arthritis and joint or muscular pain [12]. As a result, natural-source medicines with minimal or no adverse effects are becoming increasingly popular.

The *Sepia pharaonis* ink (SI), derived from the cuttlefish’s ink gland, has been used in traditional medicine for centuries. It is listed as a traditional Chinese medicine in the Compendium of Materia Medica and has been employed to treat various conditions such as heart pain and hemostasis. In traditional Indian homeopathy, it is used to treat menopausal symptoms [13], endometrial hyperplasia, uterine fibroids [14], and PCOS [15]. Though studies have found significant positive leads in treating gynecological disorders, the exact mechanism of action is not yet known. Considering the etiology of the disease and the side effects of the existing treatment strategies, the effect of SI on insulin resistance in the PCOS condition was analyzed in the present study.

## 2. Materials and Methods

### 2.1. Chemicals and Kits

The chemicals PBS (phosphate buffer saline), DHEA (dehydroepiandrosterone 5169 PQF010), metformin (D150959), sesame oil (S3547), and glucose (158968) were purchased from Sigma-Aldrich chemicals (Burlington, MA, USA); ELISA kits for testosterone (ab108666), progesterone (ab108670), and estradiol (ab108667) were from Abcam (Cambridge, UK); and the IP insulin injections used were commercially available human biphasic isophane from Eli Lilly and Company (Indianapolis, IN, USA).

### 2.2. Sepia pharaonis Ink (SI) Preparation

Fresh cuttlefish were collected from the Cochin coast, Kerala (India) and were frozen immediately. Frozen fish were dissected within 24 h and the ink glands were separated. Crude ink was collected by a milking procedure [16] and lyophilized immediately to a fine black powder and stored at −20 °C.

### 2.3. Experimental Design

Healthy C57BL/6 female mice at the age of 3–4 weeks were used in the study. Animal studies were performed at the CSIR–IICT (Council of Scientific and Industrial Research—Indian Institute of Technology) animal house facility (CCSEA registration No:97/GO/RBi/S/1999/CPCSEA). All the animals were fed with a pelleted diet which is commercially available and were given autoclaved fresh drinking water. Animals were housed in a controlled environment (20–22 °C temperature and 50–60% relative humidity) with a light/dark cycle (12 h light/12 h dark). All animal protocols were approved by IAEC (Institutional Animal Ethical Committee, Approval No: IICT/IAEC/040/2022) of CSIR–IICT, Hyderabad, India.

The PCOS mouse model was developed by treating the animals with DHEA. The mice were divided into 6 groups with n = 6 in each (Table 1). Group I was vehicle control and the mice were given only sesame oil (100 µL) subcutaneously. Group II was disease control and the animals were administered with 100 µL of DHEA (60 g/kg body weight dissolved in sesame oil) subcutaneously. Group III to Group V were the treatment groups, in which the animals were administered with different doses of SI (sepia ink suspended in PBS) orally following the administration of DHEA subcutaneously. Group III (DHEA + SI-50), receiving DHEA + 50 mg/kg body weight (wt.) of SI; Group IV (DHEA + SI-100), recieving DHEA + 100 mg/kg body wt. of SI; and Group V (DHEA + SI-200), receiving DHEA + 200 mg/kg body wt. of SI, were taken as treatment groups. Group VI animals were administered with the standard drug metformin (DHEA + 50 mg/kg body wt. of metformin dissolved in water). All the animals were treated for 28 days with the appropriate dosages according to group.

### 2.4. Measurement of Body and Ovary Weights

Body weights of all study animals were measured at an interval of 3 days from day 1 to day 28. Weight of ovaries was measured immediately after CO_2_ euthanasia.

### 2.5. Determination of Estrous Cycle Regularity

Estrous cycle regularity was checked by vaginal cytology from the 14th day of the study for 7 consecutive days during the morning. Vaginal smears of the mice were collected using a saline-dipped sterile cotton swab. Swabs were inserted into the vagina of the mouse by gentle rotation and were then smeared on to a glass slide. Air-dried smears were then observed under light microscope (Leica Microsystems, Wetzlar, Germany) after staining with methylene blue (1%). The periodicity of the estrous cycle is determined based on the presence or absence of the fraction of cornified and nucleated epithelial cells as well as leucocytes. Comparison of the irregularities of estrous cycle was done based on the hours the mice spent in each stage, using the following formula:

% of time spent in each phase = (hrs spent in each phase/total no. of hrs) × 100

% of irregularity = (no. of animals with irregularity/total no. of animals in group) × 100

### 2.6. IPGTT (Intraperitoneal Glucose Tolerance Test)

An intraperitoneal glucose tolerance test was performed on the 21st day of the study period. Animals were kept on an overnight fast on the previous night and were given 2 g/kg body wt. (of 50% solution) of glucose through IP route. Blood glucose levels were calculated at different time frames of 0, 30, 60, 90, and 120 min after the administration using a commercially available glucometer (Accu-Chek, North Ryde, Australia).

### 2.7. IPITT (Intraperitoneal Insulin Tolerance Test)

An intraperitoneal insulin tolerance test was performed on the 24th day of the study period. Animals were kept on a fast, as for the IPGTT, and were given 1 U/kg body wt. of insulin (prepared in saline) through IP mode. Blood glucose levels were calculated at different time frames of 0, 30, 60, 90, and 120 min after the administration using a commercially available glucometer (Accu-Chek).

### 2.8. Determination of Hormone Levels

The levels of testosterone, estradiol, and progesterone were determined by using commercially available ELISA (enzyme-linked immunosorbent assay) kits from Abcam (UK) as per the instruction manual provided by the manufacturer.

### 2.9. Determination of Insulin Resistance Biomarkers IGF-1, IRS-1, and AR-1

Total RNA was isolated from the ovary by triazol—chloroform method [17] and quantified using nanodrop, and cDNA for the same was synthesized using the RNeasy Cleanup Kit (Qiagen, Germantown, MD, USA). Primers for insulin resistance biomarkers IGF-1, IRS-1, and AR were designed using Primer3 software (version 0.4.0) (Table 2). Housekeeping gene GAPDH was used as a reference control for normalization. Quantitative RT-PCR was carried out using SYBR Green (Takara, San Jose, CA, USA), and the mRNA expression was studied by calculating the Ct (ΔCt) values.

### 2.10. Histopathology

Ovary and liver samples were collected, along with samples of other organs, and were stored in formalin (10% buffered) for histomorphometric and hepatic steatosis analysis, respectively. Tissue samples were embedded in paraffin wax after a series of treatments with graded alcohols. Fine sections of thickness 4 μm were made and were stained with hematoxylin and eosin (H&E) for ovaries and Mason’s trichrome (MT) for liver samples. Stained slides were assessed in a blinded way by a pathologist using a light microscope (Olympus, Tokyo, Japan).

### 2.11. Statistical Analysis

All data were analyzed statistically by one-way/two-way ANOVA followed by Dunnett’s post hoc method. Values used in analysis are expressed as mean ± SEM (n = 6), using the GraphPad Prism^®^ software (version 0.4.0) (GraphPad Software Inc., San Diego, CA, USA), and a *p* value < 0.05 was considered statistically significant.

## 3. Results

### 3.1. Determination of Body and Ovary Weights

**Figure 1:**
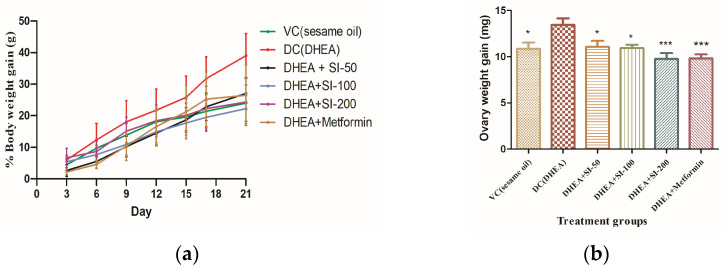
Determination of body weight and ovary weight. (**a**) Percentage of body weight change by day. (**b**) Ovarian weight changes in treatment groups and VC group (Vehicle control) in comparison with the DHEA group (DC). One-way ANOVA was used for calculating the data by applying the Dunnett post hoc test. Results were represented as mean ± SEM, n = 6. * *p* < 0.05; *** *p* < 0.001 vs. the DHEA group.

Body weight determination is a phenotypic index for PCOS. Body weight of mice was determined once every three days during the study time. Figure 1a indicates that the treatment groups with SI (all concentrations) showed reduced body weight compared to the disease control group with DHEA and on par with the metformin group by the end of the study. The weight of the ovaries was significantly increased in the DHEA (PCOS) group compared to that of the treatment groups (Figure 1b).

### 3.2. Effect of SI on Anovulation and Major Reproductive Hormones

**Figure 2:**
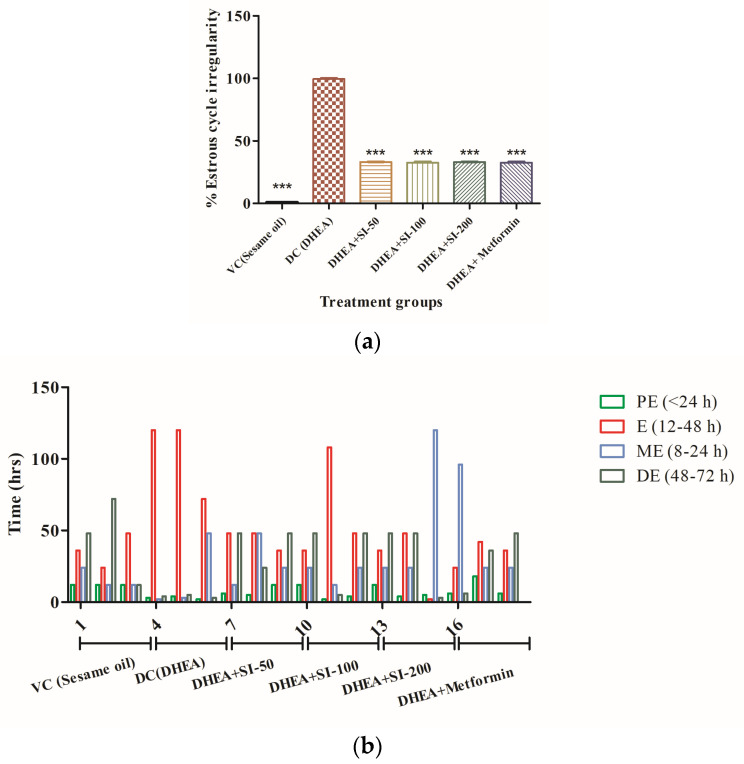

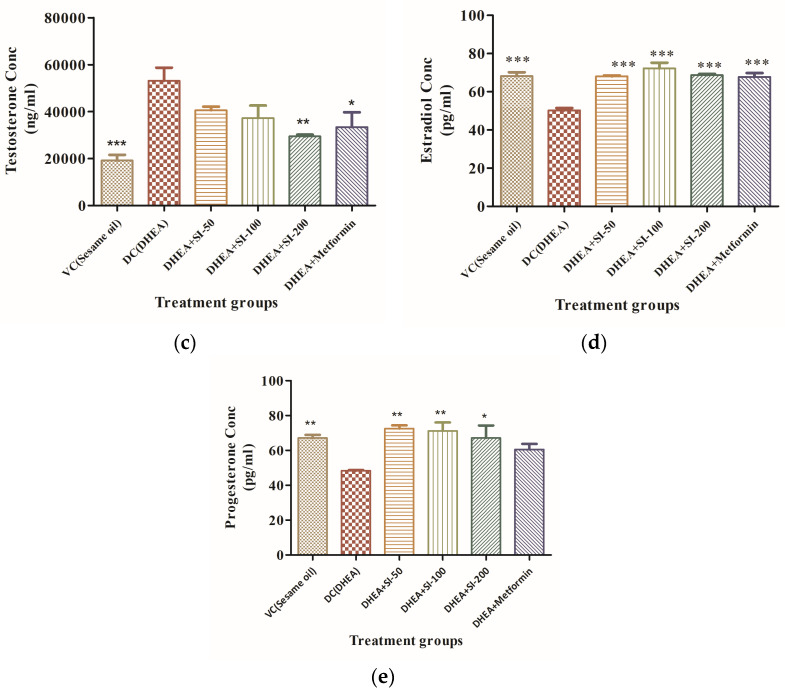
Effect of SI on anovulation and major reproductive hormones. (**a**) Percentage irregularity of estrous cycle. (**b**) Percentage of hours spent in each phase of the estrous cycle (PE—proestrus, E—estrus, ME—metestrus, DE—diestrus). (**c**) Testosterone. (**d**) Estradiol. (**e**) Progesterone. One-way ANOVA was used for calculating the data by applying the Dunnett post hoc test. Results were represented as mean ± SEM, n = 6. * *p* < 0.05; ** *p* < 0.01; *** *p* < 0.001 vs. the DHEA group.

PCOS is best described by increase in the weight of ovaries and chronic anovulation. The methylene-blue-stained vaginal smears showed the anovulatory stage and inappropriate time frames of different phases of the estrous cycle in the DHEA group. However, the irregularity is marginal and almost absent in the SI-treated groups and standard metformin group, as shown in Figure 2a,b.

Testosterone excess and decrease in the levels of estradiol and progesterone are indicators of typical PCOS. Hormonal imbalances, especially of testosterone, estradiol, and progesterone, confirmed the induction of PCOS with DHEA treatment. The SI-treated groups had decreased testosterone levels, as in the standard metformin group, and levels of estradiol and progesterone in the treatment groups and metformin group were increased significantly compared to the DHEA group (Figure 2c–e).

### 3.3. Effect of SI on Glucose Tolerance

**Figure 3:**
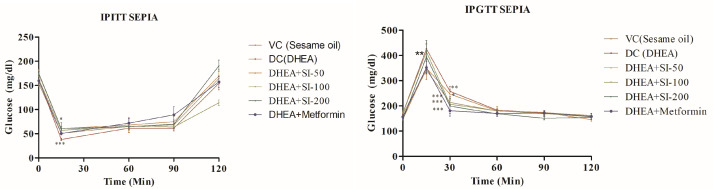
IPITT and IGTT at different time frames. One-way ANOVA was used for calculating the data by applying the Dunnett post hoc test. Results were represented as mean ± SEM, n = 6. * *p* < 0.05; ** *p* < 0.01; *** *p* < 0.001 vs. the DHEA group.

Plasma glucose levels were studied to understand the intensities of glucose tolerance and insulin resistance in all the animals by performing IPGTT and IPITT. In all groups other than DHEA (disease control), glucose levels successfully reverted to normalcy after 120 min (Figure 3).

### 3.4. Effect of SI on Ovary and Liver Histopathology

**Figure 4:**
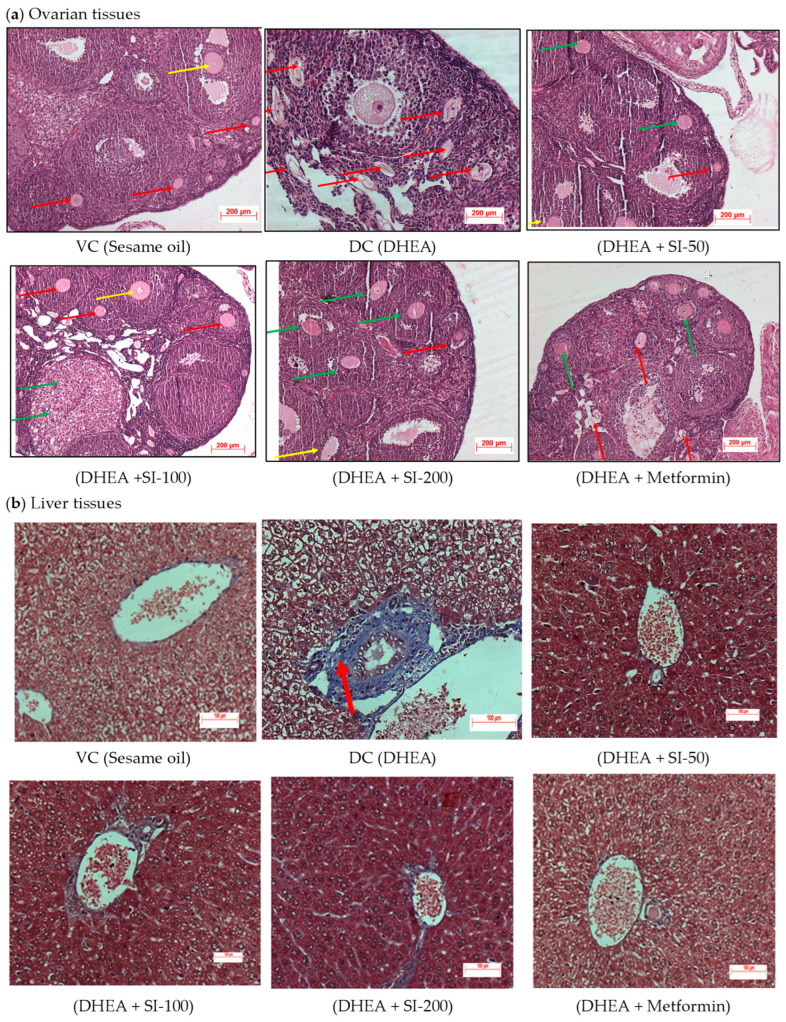
Histopathology of ovarian and liver tissues. (**a**) Histopathology of H&E-stained ovarian tissues. Multi-focal severe cystic degeneration with accumulation of fluids and atrophy was observed in primordial follicles (red arrow), secondary follicles (green arrow), and territory or antral follicles (yellow arrow) in the cortex region of the ovary in the PCOS (DC) group. Mild degeneration of normal follicles was observed in the treatment groups. (**b**) Histopathology of MT-stained liver tissues. Moderate fibrosis—bluish coloration of fibrous tissue with brownish nucleus (red arrow)—was observed in the peribiliary region and periportal region of the liver in the DC group, and the liver tissue appeared normal in all the treatment groups. The study was done with n = 3 for both the tissues.

Histopathological studies of the ovary have revealed the presence of atretic follicles, cystic degeneration, and accumulation of fluids in the DC group. H and E stained ovarian tissues (Figure 4a) showed mild cystic degeneration in the SI-50 treatment group, a moderate effect on normal ovarian follicles in the SI-100 group, and corpus luteum in the SI-200 treatment group. However, the histopathology of the SI-200 treatment group was on par with that of the metformin group.

Liver histopathology of all the treatment groups and VC showed no fibrosis and normal portal lobules (Figure 4b), but the DHEA-treated PCOS group showed moderate fibrosis in the peribiliary and periportal regions of the liver.

### 3.5. Effect of SI on Expression of Genes Related to Insulin Resistance

**Figure 5:**
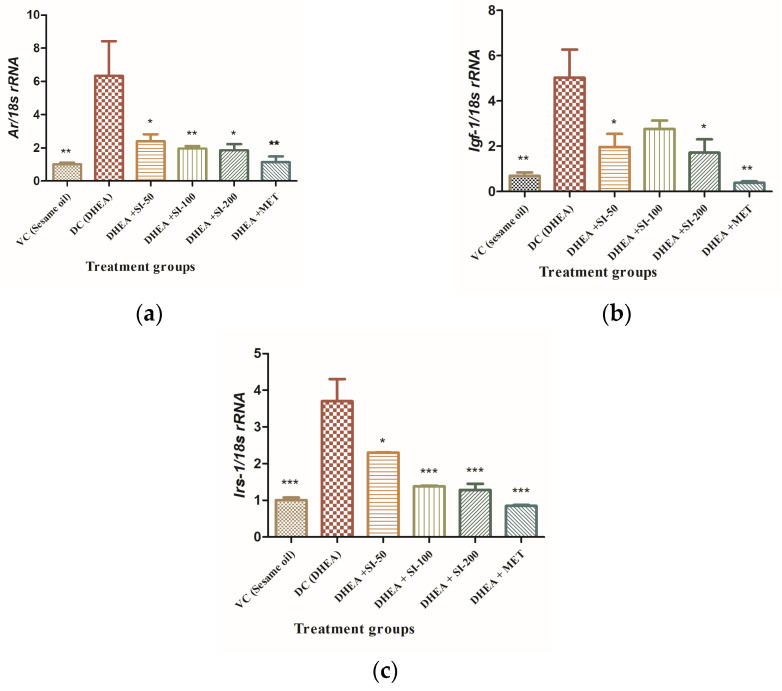
mRNA expression analysis of genes from ovarian tissue. (**a**) *Ar1* gene. (**b**) *Igf1* gene. (**c**) *Irs1* gene. One-way ANOVA was used for calculating the data by applying the Dunnett post hoc test. Results were represented as mean ± SEM, n = 6. * *p* < 0.05; ** *p* < 0.01; *** *p* < 0.001 vs. the DHEA group.

SI treatment exhibited significant reduction in mRNA expression of androgen receptor (*Ar1*), insulin-like growth factor 1 (*Igf1*), and insulin receptor substrate1 (*Irs1*) genes in a dose-dependent manner (Figure 5). Though the DHEA + SI-50 group showed a mild decrease in expression of the *Irs1* gene, it showed a significant effect on *Ar1* and *Igf1* expression. The effect of SI is on par with standard metformin, which is a potent drug in the treatment of diabetes.

## 4. Discussion

PCOS is correlated with many metabolic disorders such as hyperandrogenism and insulin resistance, influencing the quality of life and long-term health of patients. So far, no treatment has satisfactorily controlled PCOS. In this research, we investigated the therapeutic effects of sepia ink and tried to explore the underlying molecular mechanisms. Our selection of the doses 50 mg/kg/day, 100 mg/kg/day, and 200 mg/kg/day of ink was based on our preliminary results and on the efficacy of these doses in previous studies [18]. Reflecting its various potent anti-inflammatory, anti-ulcerogenic, and anti-cancer properties [19], our findings showed that the ink was beneficial in DHEA-induced PCOS conditions, indicating its potential role in the treatment of PCOS-related reproductive and metabolic disorders, such as hyperandrogenism, and as a potential candidate against hyperinsulinemia.

In the present study, DHEA was used to induce polycystic ovary syndrome in mice. Previous reports suggest that a DHEA-induced PCOS condition is similar to human PCOS in many ways. The working of this model was confirmed by regular examination of vaginal smears and the presence of persistent vaginal cornification. As evidenced, there was a marked increase in testosterone levels when compared to control animals, indicating the hyperandrogenic status of the PCOS condition.

Sepia ink was able to normalize serum testosterone levels similarly to metformin. Serum levels of progesterone and estradiol were decreased in the PCOS-induced group. Decreased progesterone levels are also indicative of anovulation, and the ink successfully restored its level to normal. A low estradiol concentration in PCOS-induced animals was significantly increased by administration of ink at different doses. The histopathology studies of treatment groups showed a significant reduction in the morphology of PCOS ovaries. Presences of antral follicles without poly-cysts were observed in treatment groups, on par with the vehicle control and standard drug metformin group.

Receptors of insulin are distributed widely in ovarian cells. Insulin has a direct effect on the production of steroids, thus playing a significant role in the regulation of ovulation [20,21]. Insulin acts on the thecal cells of the ovary, where androgen production is prominent, thus increasing androgen synthesis [22]. The effect of high insulin on anovulatory granulosa cells of PCOS leads to premature differentiation and stagnation of follicular growth [23]. It was shown that the expression of insulin receptors and molecules involved in the insulin signaling pathway was adversely affected in PCOS, leading to reproductive disorders [24].

Hyperandrogenism, too, plays a significant role in the insulin signaling pathway by feedback loops and regulation of the *Irs*1 gene [25]. From the perspective of gene expression in our study, sepia ink has been found to downregulate the mRNA expression of *Ar* (androgen receptor), *Igf-*1 (insulin-like growth factor 1), and *Irs-*1 (insulin receptor substrate 1) in the ovary. In PCOS, the androgen receptor (AR) plays a crucial role in mediating the effects of androgens on ovarian function. Our study has shown increased *AR* mRNA expression in the ovaries of the DHEA group compared to controls. This observation aligns with previous research by Dey et al., [26] which demonstrated a significant rise in AR expression in letrozole-treated rats. Additionally, Manneras et al. [27] noted *Ar* upregulation linked to endogenous testosterone accumulation, leading to ovarian alterations. In PCOS mice treated with sepia ink, a decrease in *Ar* mRNA expression was observed, indicating the ink’s anti-androgenic effects. This suggests that sepia ink may help alleviate the hyperandrogenic state associated with PCOS by reducing *Ar* expression in the ovary. Similarly, the decrease in *Igf-1* and *Irs-1* mRNA expression observed in SI-treated samples suggests its potential to enhance insulin sensitivity and regulate ovarian function; this reduction in expression levels could help alleviate symptoms associated with PCOS, such as insulin resistance and hormone imbalance, offering a promising therapeutic intervention for PCOS.

The liver is the main site for promotion of glycogenesis, inhibition of gluconeogenesis, and insulin clearance [28]. It is clear that IR and hyperinsulinemia directly affect the synthesis of the IGF-1 binding protein, which eventually results in increased circulating concentration of IGF-1. This triggers the ovarian cells to produce more androgens [29]. Long-term excess androgen cause chronic liver diseases, and its prevalence in PCOS have increased significantly in recent years [30]. Many human studies also confirmed IR as a risk factor for liver diseases in PCOS conditions [31]. Testosterone, dihydrotestosterone, and dehydroepiandrosterone (DHEA) are androgenic hormones that promote apoptosis in peripheral cells like hepatocytes. The excess production of these androgens in polycystic ovary syndrome creates a pro-apoptotic environment that may directly advance liver disease [32]. Studies have shown that non-alcoholic fatty liver disease (NAFLD) is more common in womenfemales with polycystic ovary syndrome (PCOS) than in those without PCOS. Specifically, the prevalence of NAFLD in PCOS patients has been found to range from 36% up to as high as 70% [33,34,35]. The MT staining of liver results in treatment groups has shown no cirrhosis condition, which is very conspicuous in the disease group, confirming its non-toxic activity in the liver. Previous studies on DHEA-induced PCOS murine models also reported mild to moderate liver fibrosis during the disease condition [36,37]. The IPGTT levels, which were higher in the DHEA group, were significantly lower in the treatment group and were on par with the standard metformin group. IPITT levels, which were less in the disease group, were higher in the treatment group, indicating that the glucose metabolism of SI-treated animals was able to return to normalcy.

The standard drug metformin used for the treatment of diabetes is also prescribed for PCOS, as it restores endometrial function by regulating IR expression [38]. However, the use of metformin has been limited due to its gastrointestinal side effects [39]. Given the results of the present study, SI targeting of the IR in PCOS might emerge as a better alternative to address this metabolic disorder.

## 5. Conclusions

Considering the face validity of the disease, different parameters like body weight, estrous cycle irregularity, insulin resistance, histopathology of ovaries, and major affected hormone levels were analyzed. Though the predictive validity of the study was aimed toward graded response, the quantal response of drug activity was observed for obvious reasons. However, a graded response was observed in parameters like body weight, estrous cycle irregularity, and insulin resistance. A quantal response was shown in the variation of the hormonal-level parameter. Concisely, all the doses showed efficacy, with varied but better effectiveness in comparison to the metformin, with no side effects. Understanding the precise mechanism of action of SI will provide greater insight in exploring specific drug targets in PCOS and other gynecological conditions.

## Figures and Tables

**Table 1:** Experimental groups and treatment concentrations.

		Induction	Treatment
**Group I**	Vehicle control (VC)		Sesame oil
**Group II**	DHEA group (DC)	DHEA in Sesame oil	
**Group III**	SI-50	DHEA in Sesame oil	SI-50 mg/Kg body wt.
**Group IV**	SI-100	DHEA in Sesame oil	SI-100 mg/Kg body wt.
**Group V**	SI-200	DHEA in Sesame oil	SI-200 mg/Kg body wt.
**Group VI**	Metformin	DHEA in Sesame oil	MET-50 mg/Kg body wt.

**Table 2:** Primer sequences for quantitative RT PCR.

Gene Name	Forward	Reverse
*Igf1*	GTGGATGCTCTTCAGTTCGTGTG	TCCAGTCTCCTCAGATCACAGC
*Irs1*	TGTCACCCAGTGGTAGTTGCTC	CTCTCAACAGGAGGTTTGGCATG
*Ar*	GGCGGTCCTTCACTAATGTCAACT	GAGACTTGTGCATGCGGTACTCAT
*GAPDH*	CATCACTGCCACCCAGAAGACTG	ATGCCAGTGAGCTTCCCGTTCAG

## Data Availability

The dataset generated and/or analyzed during the current study are not publicly available but are available from the corresponding author on request.
