# Correction: Yamarthi et al. *Sepia pharaonis* Ink Mitigates Dehydroepiandrosterone-Induced Insulin Resistance in Mouse Model of Polycystic Ovarian Syndrome. *Pathophysiology* 2024, *31*, 408–419

**DOI:** 10.3390/pathophysiology32010004

**Published:** 2025-01-20

**Authors:** Prathyusha Yamarthi, Rama Satyasri Kotipalli, Samatasai Patnaik, Kv Veena, Muralidharan Kathirvel, Rajkumar Vutukuri, Manjula Bhanoori

**Affiliations:** 1 Department of Biochemistry, Osmania University, Hyderabad 500007, India; prathyusha.yamarthi@gmail.com (P.Y.); veenathareesh090@gmail.com (K.V.); 2 Applied Biology, CSIR—Indian Institute of Chemical Technology, Hyderabad 500007, India; satyasri.15247@csiriict.in (R.S.K.); samata.spatnaik@gmail.com (S.P.); muralidharan@iict.res.in (M.K.); 3 Institute of General Pharmacology and Toxicology, Pharmazentrum Frankfurt, Goethe University Frankfurt, 60596 Frankfurt am Main, Germany

In the original publication [1], there was a mistake in Figure 4 as published. The panels DHEA+SI-50 and DHEA+SI-100 in Figure 4A presented an overlap due to the faulty arrangement of the images. The corrected Figure 4 appears below. The authors state that the scientific conclusions are unaffected. This correction was approved by the Academic Editor. The original publication has also been updated.

## Figures and Tables

**Figure 4:**
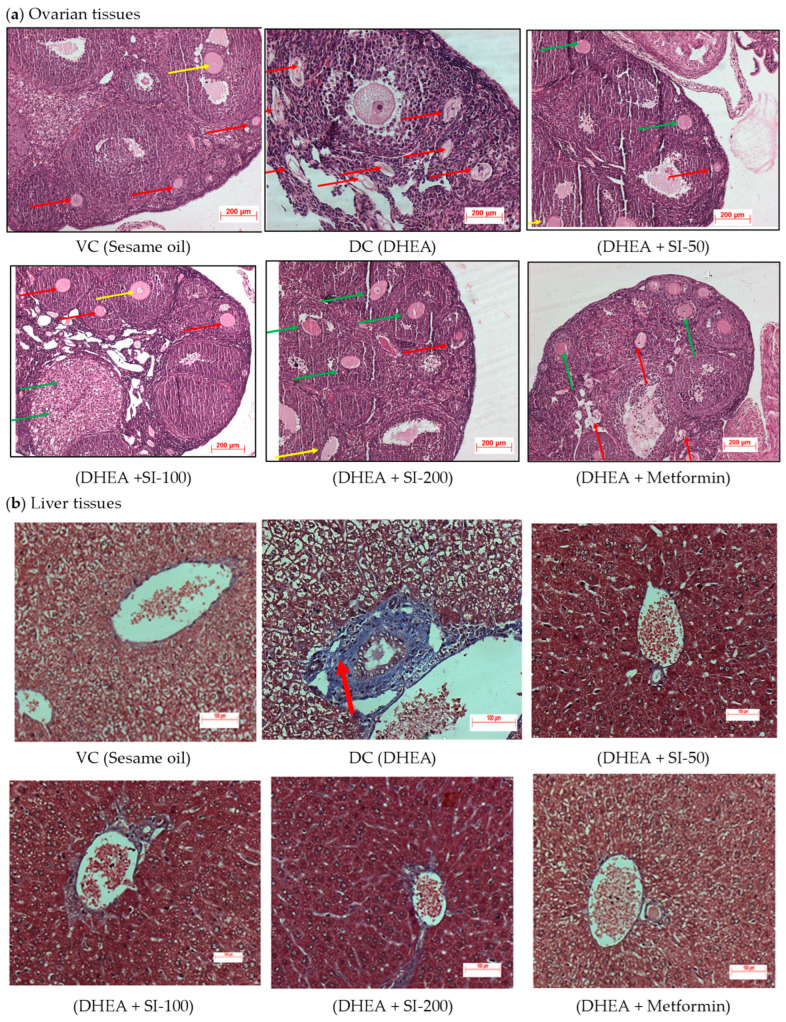
Histopathology of ovarian and liver tissues. (**a**) Histopathology of H&E-stained ovarian tissues. Multi-focal severe cystic degeneration with accumulation of fluids and atrophy was observed in primordial follicles (red arrow), secondary follicles (green arrow), and territory or antral follicles (yellow arrow) in the cortex region of the ovary in the PCOS (DC) group. Mild degeneration of normal follicles was observed in the treatment groups. (**b**) Histopathology of MT-stained liver tissues. Moderate fibrosis—bluish coloration of fibrous tissue with brownish nucleus (red arrow)—was observed in the peribiliary region and periportal region of the liver in the DC group, and the liver tissue appeared normal in all the treatment groups. The study was done with n = 3 for both the tissues.
